# Effect of Green and Brown Propolis Extracts on the Expression Levels of microRNAs, mRNAs and Proteins, Related to Oxidative Stress and Inflammation

**DOI:** 10.3390/nu9101090

**Published:** 2017-10-01

**Authors:** Vincenzo Zaccaria, Valeria Curti, Arianna Di Lorenzo, Alessandra Baldi, Cristina Maccario, Sabrina Sommatis, Roberto Mocchi, Maria Daglia

**Affiliations:** 1 Department of Drug Sciences, Medicinal Chemistry and Pharmaceutical Technology, University of Pavia, Via Taramelli 12, 27100 Pavia, Italy; vincenzo.zaccaria01@universitadipavia.it (V.Z.); valeria.curti86@hotmail.it (V.C.); arianna.dilorenzo01@universitadipavia.it (A.D.L.); alessandra.baldi@outlook.it (A.B.); 2 Department of Molecular Medicine, University of Pavia, Via Forlanini 6, 27100 Pavia, Italy; cristina.maccario01@gmail.com (C.M.); sabrina.sommatis01@universitadipavia.it (S.S.); marketing@ub-careitaly.it (R.M.)

**Keywords:** brown and green propolis, anti-inflammatory activity, antioxidant activity, microRNAs, mRNAs, proteins

## Abstract

A large body of evidence highlights that propolis exerts many biological functions that can be ascribed to its antioxidant and anti-inflammatory components, including different polyphenol classes. Nevertheless, the molecular mechanisms are yet unknown. The aim of this study is to investigate the mechanisms at the basis of propolis anti-inflammatory and antioxidant activities. The effects of two brown and green propolis extracts—chemically characterized by RP-HPLC-PDA-ESI-MSn—on the expression levels of miRNAs associated with inflammatory responses (miR-19a-3p and miR-203a-3p) and oxidative stress (miR-27a-3p and miR-17-3p), were determined in human keratinocyte HaCat cell lines, treated with non-cytotoxic concentrations. The results showed that brown propolis, whose major polyphenolic components are flavonoids, induced changes in the expression levels of all miRNAs, and was more active than green propolis (whose main polyphenolic components are hydroxycinnamic acid derivatives) which caused changes only in the expression levels of miR-19a-3p and miR-27a-3p. In addition, only brown propolis was able to modify (1) the expression levels of mRNAs, the target of the reported miRNAs, which code for Tumor Necrosis Factor-α (TNF-α), Nuclear Factor, Erythroid 2 Like 2 (NFE2L2) and Glutathione Peroxidase 2 (GPX2), and (2) the protein levels of TNF-α and NFE2L2. In conclusion, brown and green propolis, which showed different metabolite profiles, exert their biological functions through different mechanisms of action.

## 1. Introduction

Propolis, also known as bee glue, is a complex mixture, produced by bees, mainly *Apis mellifera* L. It contains resin and other materials (lipophilic material from leaves, mucilage, gum, latex) collected by bees from various botanical sources—plant leaves, buds and exudates—which are digested by bee saliva enzymes and mixed with beeswax.

About 50% of propolis is comprised of plant resin, with beeswax accounting for approximately a further 30%. Of the remainder, about 10% is essential oils, 5% is pollen and 5% is other organic substances. Propolis also contains vitamins, including B1, B2, B6, C and E, and amino acids, derived from the bees’ metabolisms [1,2]. Propolis also contains some mineral elements, such as Mg, Ca, I, K, Na, Cu, Zn, Mn and Fe [3], and heavy metals, such as Cd, Hg, and Pb [4]. The first studies on the chemical characterization of propolis date back to the beginning of the 20th century [5]. With progress being made in analytical methods, more than 300 compounds have been identified in propolis to date, including flavonoids, terpenoids, phenolic acids and phenolic esters and sugars. 

As far as biological activities are concerned, there are hundreds of studies present in the scientific literature supporting the healthy properties of propolis, such as gastroprotective, hepatoprotective [6,7], immunomodulatory [8], wound healing [9,10], antidiabetic [11,12], and antineoplastic properties [10]. These are ascribed to the three main activities of propolis, namely antioxidant [13], anti-inflammatory [8], and antimicrobial [14] activities.

One of the most studied properties of propolis is its antioxidant capacity [10]. The main compounds responsible for this activity are caffeoylquinic acid derivates, which show higher radical scavenging activity than most common antioxidants, such as vitamin C and vitamin E. In addition, caffeic acid phenethyl ester (CAPE) exerts protective effects on the lipid peroxidation of erythrocyte membranes [15]. The strong antioxidant activity of propolis suggests that it could be used as an ingredient in the preparation of functional foods and food supplements, and may be useful in the prevention and dietary management of patients with chronic diseases caused by oxidative stress [13]. For instance, in 2004, Lahouel et al. found that propolis can also have protective effects against drug side effects and cancer chemotherapeutic agent toxicity. They studied the effects of oral administration of propolis extract (60 mg/kg), for 14 days, in Wistar rats treated with cyclophosphamide, vinblastine and paracetamol—three chemotherapeutic drugs with high hepatotoxic activity, which deplete hepatic glutathione and induce lipoperoxidation. Rats supplemented with propolis had clearly lower drug toxicity effects following the treatment, likely owing to the action of propolis flavonoids on glutathione and glutathione-S-transferase turnover, which permitted the capture of reactive metabolites from the chemotherapeutic drugs [16].

As an anti-inflammatory agent, propolis has been shown to inhibit the synthesis of prostaglandins, activate the thymus, help the immune system by promoting phagocytic activity, stimulate cellular immunity and improve curative effects in epithelial tissues [8]. Based on literature data, CAPE blocks the release of interleukin 1β (IL-1β) through the inhibition of Nuclear Factor kB (NF-kB) activity. Propolis flavonoids and CAPE have been compared to the cyclooxygenase (COX) inhibitor, indomethacin (IM), and the lipoxygenase (LOX) inhibitor, nordihydroguaiaretic acid (NDGA), and were found to have the same effects as IM and NDGA [17]. In addition, a study showed that CAPE inhibits the release of inflammatory cytokines and simultaneously increases the production of anti-inflammatory cytokines, such as IL-10 and IL-4. The same research showed that CAPE decreases the infiltration of inflammatory cells, such as neutrophils and monocytes [18].

In regard to epigenetic mechanisms, microRNAs (miRNAs) play a very important role in the regulation of gene expression. They are a class of endogenous non-coding RNA, consisting of about 22 nucleotides, which are able to regulate gene expression at the post-transcriptional level. They exert their functions by binding complementary sequences on messenger RNA (mRNA) targets, interfering with the translation process and preventing or altering gene expression [19].

There are some studies on the epigenetic effects of propolis in the current scientific literature. In a 2014 research article, Kumazaki et al. showed that two propolis cinnamic acid derivatives, baccharin and drupanin, induce apoptosis in human drug-resistant colon cancer cells by increasing the expression level of anti-oncogenic miR-143, which leads to down-regulation of its target gene, Erk5, and consequently contributes to cell cycle arrest [20]. Similar findings were provided by Cuevas et al., who observed an attenuation in atherosclerotic lesions in low density lipoprotein receptor gene knockout mice, which was related to an overexpression of miR-181a, miR-106a and miR-20b, which are involved in the modulation of pro-angiogenic factors [21]. In 2015, a study on Chilean propolis corroborated the epigenetic effect of this product by showing an overexpression of miR-19b, which targets the mRNA coding for proangiogenic proteins, in human endothelial cells [22].

Despite a great number of reports on the antioxidant and anti-inflammatory properties of propolis, its molecular mechanisms are, as yet, unknown. Thus, the aim of this study is to investigate the effects of two types of propolis extracts—characterized by RP-HPLC-PDA-ESI-MSn—on the expression levels of miRNAs, mRNAs and proteins associated with oxidative stress and inflammatory responses in human keratinocyte HaCat cell lines. These were selected because they represent the most abundant cell type in the epidermis, and are highly present in the oral cavity—the main route for administration of propolis products. 

In this research, we have particularly focused our studies on propolis from Brazil, which is characterized by a green color, and propolis from Europe, which is characterized by a brown color. The green tint of Brazilian propolis is a consequence of its botanical origin, as Brazilian bees primarily collect resin from the young plant tissues of *Baccharis dracunculifolia* DC, a shrub in the Asteraceae family, widespread in southern Brazil, which contains a high concentration of chlorophyll [23]. European propolis is principally derived from resins of the *Populus* species (*P. alba*, *P. nigra*, *P. tremula*) [24].

## 2. Materials and Methods

### 2.1. Sample Preparation

In order to evaluate the activity of green and brown propolis samples in miRNA transcription, a sample of hydroglyceric extract was prepared.

This sample was obtained using the Multi Dynamic Extraction (MED) method [25], as recorded by B Natural S.r.l., a company which delivers and prepares propolis samples, using water–alcohol extraction, with varying concentrations of alcohol, thus allowing for the extraction of all polyphenolic compounds present in the raw propoevolis material, regardless of their varying levels of solubility. The concentrate thus obtained was subsequently dissolved in glycerin. The MED^®^ method allows the elimination of inactive resins, leading to the production of a complex concentrate, which is rich in polyphenols and in the glycosylated polyphenolic fraction in particular. This process includes several steps for the preparation of polyphenol-rich propolis whole extracts. These consist of an initial aqueous extraction from dewaxed raw propolis, a series of extractions on the residue using an ethanol/water mixture, with each extraction being carried out on the residue from the preceding extraction using a higher degree of alcohol, and finally the combination of water and water–ethanol extracts, with optional concentration or formulation steps to prepare for further testing.

The raw propolis samples were processed as follows:

- Aqueous extraction, to remove waxes and impurities from raw materials, using a 1:1 solvent/propolis ratio, at 80 °C for 10 h and with 100 Watt ultrasounds. After cooling at 8 °C, the solution was filtered with a 30 μm filter.
- Three hydro-alcoholic extractions, one for each insoluble residue of the preceding extraction step, carried out using different alcoholic degrees and temperatures, from 4 to 36 h, with a fixed 1:1 solvent/propolis residue ratio. Each extraction step was followed by a sample cooling step at circa 15 °C, a filtration step with a 30 to 50 μm filter and a concentration step using a rotating evaporator, to obtain a soft mixture.
  (1) The first extraction step used a water/ethanol mixture with an alcoholic concentration ranging from 35 to 40 alcoholic degrees, at 50 °C;
  (2) The second extraction step used a mixture with an alcoholic concentration ranging from 55 to 60 alcoholic degrees, at 70 °C;
  (3) The third extraction step used a mixture with an alcoholic concentration ranging from 70 to 80 alcoholic degrees, at 80 °C.
- Concentration: the combined extracts were mixed and concentrated to a residual humidity value ranging from 15 to 20% by weight.
- Dissolution in glycerin: the concentrated extracts were evaporated to remove ethanol. The dough was mixed with hot glycerin and water for 2 h within a mixer, and then cooled at 10 °C to give a non-alcoholic liquid. After precipitation the solution was filtered twice, using a 30 μm and a 10 μm filter.

### 2.2. Analyses of Propolis Extracts (RP-HPLC-PDA-ESI-MSn)

The chromatographic analyses were performed by means of the RP-HPLC-PAD-ESI-MSn method, set up by Cui-ping et al., with some modifications [26]. The chromatographic analyses were performed using a Thermo Finnigan Surveyor Plus HPLC, equipped with a quaternary pump, a Surveyor UV-Vis diode array detector and a LCQ Advantage ion trap mass spectrometer (Thermo Fisher Scientific, Waltham, MA, USA). Xcalibur software (2.0 SR2, Thermo Fisher Scientific, Waltham, MA, USA) was used to control the HPLC instrumentation and to analyze the data. Compound separation was obtained with an analytical Synergi Fusion RP-18 column (150 × 4.6 mm, 5 μm), equipped with a Hypersil Gold C18 precolumn (10 × 2.1 mm, 5 μm), all produced by Phenomenex (Torrance, CA, USA). The mobile phase used was acidified water, with 0.1% formic acid (eluent A) and methanol (eluent B). The run time was 110 min in total, including the reconditioning of the column. The flow rate was maintained at 1.00 mL/min, and the temperatures of the autosampler and column were kept at 4 and 33 °C. The volume of injection was set to 5 μL. The elution method is described in Table 1. Chromatograms were registered at 5 different wavelengths (λ), equal to 254, 280, 330, 370 and 395 nm. The HPLC-ESI-MSn data were collected using Xcalibur software under a negative ionization mode. For this purpose, the ion trap was set in full scan mode to detect all mass-to-charge ratios (*m*/*z*) in the selected range, data dependent scan, and MSn mode, in order to obtain further discrimination between compounds.

### 2.3. Cell Culture

Human keratinocyte cell lines (HaCaT, code BS CL 168) from the IZSLER Institute (Instituto Zooprofilattico of Lombardy and Emilia Romagna) were selected for this study. Dulbecco's Modified Eagle Medium (D-MEM) High-Glucose was used as the culture medium (complete medium), supplemented with 10% fetal bovine serum (FBS), 1% l-glutamine (2 mM) and antibiotics (penicillin, 100 IU/mL and streptomycin, 100 μg/mL). Cells were grown in sterile conditions and kept at 37 °C in an atmosphere with 5% carbon dioxide.

### 2.4. Cell Viability Test

Preliminary experiments, using an MTT (3-(4,5-dimethylthiazol-2-yl)-2,5-diphenyltetrazolium bromide) assay, have allowed us to identify non-cytotoxic concentrations of propolis. HaCat cells were seeded in 96-well plates, at a density of 1.5 × 10^4^ cells per well and incubated at 37 °C with 5% CO_2_. The HaCat cells were treated with propolis extracts 24 h after seeding. The extract was weighed and dissolved in complete culture medium, at a concentration of 25 mg/mL. Subsequent serial dilutions (1:2) were prepared to reach the final concentration of 0.019 mg/mL. The control solution was prepared using 90% glycerol (from B Natural S.r.l.) and 10% H_2_O, and was tested under the same conditions, in order to exclude its direct cytotoxicity. The treatments were performed for 24 h. At the end of this period, and after morphological observation under a microscope, 10 μL of the stock 5 mg/mL of MTT in phosphate-buffered saline (PBS) were added to the HaCat cells, for 2 h, at 37 °C. At the end of the incubation period, after removal of the culture medium and washing with PBS, cells were added to 100 μL of DMSO to solubilize the formazan crystals. Spectrophotometric readings were then carried out at a wavelength of 570 nm. Cell viability was calculated by measuring the optical densities of treated samples compared to control samples (cells plus glycerol). Each value given in the results represents the mean ± standard deviation of three independent experiments, each consisting of three replicates [27].

### 2.5. Cell Treatment with Green and Brown Propolis

HaCat cells were seeded in Petri dishes at a density of 1.5 × 10^6^, for 24 h. Cells were treated for a further 24 h with the first three non-cytotoxic concentrations of propolis extracts: 3.125 mg/mL, 1.56 mg/mL, 0.78 mg/mL. Untreated cells were used as controls. Cells were collected and counted at the end of the incubation period, according to the standard protocol, which includes a brief wash in PBS to eliminate the supernatant. The resulting pellets were stored at −80 °C.

### 2.6. RNA Extraction and miRNA Real-Time PCR

Total RNA was extracted from the cell pellets using the miRNAeasy Mini Kit Qiagen, according to manufacturer’s instructions [28].

Quantitative RNA analyses were performed using a fluorometric method with a Qubit tool (Invitrogen, CA, Grand Island, NY, USA), using the Quant-iT RNA Assay Kit (sensitivity from 5 to 100 ng) with the following protocol: 2 μL of RNA were added to 200 μL of a “working solution”, obtained by mixing 1 μL of Qubit RNA reagent with 199 μL of Qubit RNA buffer. The quantification was performed following calibration of the instrument using appropriate standards (0 and 10 ng/mL).

The total RNA was retro transcribed to a DNA copy (cDNA) using the miRCURY LNATM Universal RT microRNA PCR Kit. This reaction only targets mature miRNA from the total RNA pool. The retro transcription protocol is as follows: 4 μL of total RNA (5 ng/L) was added to 4 μL of 5 × reaction buffer, 2 μL of enzyme mix, 1 μL of synthetic spike-in and 9 μL of nuclease free water. The mixture was then incubated in a thermocycler (SureCycler 8800-Agilent Technologies, Cernusco sul Naviglio, Milano, Italy) at 42 °C for 1 h, then 95 °C for 5 min and then immediately cooled to 4 °C.

In order to evaluate the expression of miRNAs, RT-PCR reactions were set up using the EcoTM Real-Time PCR System (Illumina, Milano, Italy) instrument and the Universal cDNA Synthesis Kit and SYBRR Green Master Mix. The PCR reaction was performed with a volume of 10 μL, containing 4 μL of cDNA, diluted 1:80, 5 μL of SYBRR Green Master Mix, and 1 μL of miR-19a-3p, 17-3p, 27a-3p, 203a-3p probes, provided by Euroclone (Pero, Milano, Italy). The reaction conditions were as follows: a first step at 95 °C for 10 min, 45 amplification cycles at 95 °C for 10 seconds, followed by a step at 60 °C for 1 min. The U6 small nuclear RNA (snU6) was used to normalize the expression data of miRNAs and each assay was performed in triplicate. To evaluate the levels of mRNA, coding for TNF-, NFE2L2, MnSOD, GPX2 and TrxR2, RT-PCR reactions were performed with the AriaMX Real Time PCR System, using Brilliant III Ultra-Fast SYBR^®^ Green RT-PCR Master Mix (Agilent, Cernusco sul Naviglio, Milano, Italy), according to the manufacturer’s protocol. Primers were designed using Primer-BLAST software (available online on 10 July 2017 at http://www.ncbi.nlm.nih.gov/tools/primer-blast). The sequences for the used primers were:

TNF-α forward: 5′-CATCCAACCTTCCCAAACGC-3′

TNF-α reverse: 5′-CTGTAGGCCCCAGTGAGTTC-3′

NFE2L2 forward: 5′-CAGTCAGCGACGGAAAGAGT-3′

NFE2L2 reverse: 5′-ACGTAGCCGAAGAAACCTCA-3′

MnSOD forward: 5′-AAACCTCAGCCCTAACGGTG-3′

MnSOD reverse: 5′-CCAGGCTTGATGCACATCTTA-3′

GPx2 forward: 5′-GAGGTGAATGGGCAGAACGA-3′

GPx2 reverse: 5′-CTCTGCAGTGAAGGGGACTG-3′

TNF-α forward: 5′-CCTCTCTGCCATCAAGAGCC-3′

TNF-α reverse: 5′-TTGAGTAACTTCGCCTGCGT-3′

TRXR2 forward: 5′-CCCTATCCCAGTGTTCCACC-3′

TRXR2 reverse: 5′-AAGGTTCCACGTAGTCCACC-3′

### 2.7. Protein Analyses

The analyses of protein expression levels were performed using an Enzyme-Linked Immunosorbent Assay (ELISA) with the microplate reader FLUOstar^®^ Omega by BMG Labtech (BMG Labtech, Ortenberg, Germany). TNF-α, NFE2L2, GPX2, Manganese Superoxide Dismutase (MnSOD) and Thioredoxin Reductase 2 (TRXR2) were quantified in the culture supernatants using a Cloud-Clone Corp Kit (respectively SEA133Hu, SEL947Hu, SEC993Hu, SES134Hu and SED376Hu kits) in 96-well plates (Cloud-Clone Corp, Houston, TX, USA)

In brief, the culture supernatant was added to 100 μL of standard diluent, and then incubated for 1 h at 37 °C. After the removal of liquid, 100 μL of detection reagent A was added and incubated for 1 h at 37 °C. Plates were then washed with 350 μL of wash solution 3 times. Next, 100 μL of detection reagent B was added and incubated for 30 min at 37 °C. After adding horseradish peroxidase-conjugated monoclonal antibodies, with the detection reagents A and B, and following a repeat wash process for a total of 5 times, samples were incubated with 90 μL of colorimetric substrate solution (3,3′,5,5′-tetrametylbenzidine) for 20 min at 37 °C, and then 50 μL of stop solution was added. Finally, a colorimetric measurement was conducted at 450 nm.

### 2.8. Statistical Analyses

The cellular effects of the treatments were tested using mixed models, in which treatments with different concentrations of a substance were considered to be a fixed effect, and the sending cell culture was considered to be a random effect. Significant values were taken to be *p* < 0.05.

Statistical analyses of quantification cycle (Cq) values were carried out using software R (ver. 3.0.3, R e2sCore Team, 2014) (Vienna, Austria). Differences between group means were estimated using a one-way analysis of variance (ANOVA), followed by Tukey’s post hoc test, with measurements of *p* < 0.05 being taken as significant.

## 3. Results

To evaluate the potential effects of propolis on the expression of miRNAs associated with oxidative stress and inflammatory processes, the human keratinocyte cell line HaCat was treated with chemically characterized green and brown hydroglyceric propolis extracts, obtained as reported in the Materials and Methods section.

### 3.1. Propolis Extracts RP-HPLC-PDA-ESI-MSn Analyses

RP-HPLC-PAD-ESI-MSn analyses of the propolis extracts led to the identification of 16 compounds in each propolis sample, as shown in Table 2 and Table 3.

**Figure 1:**
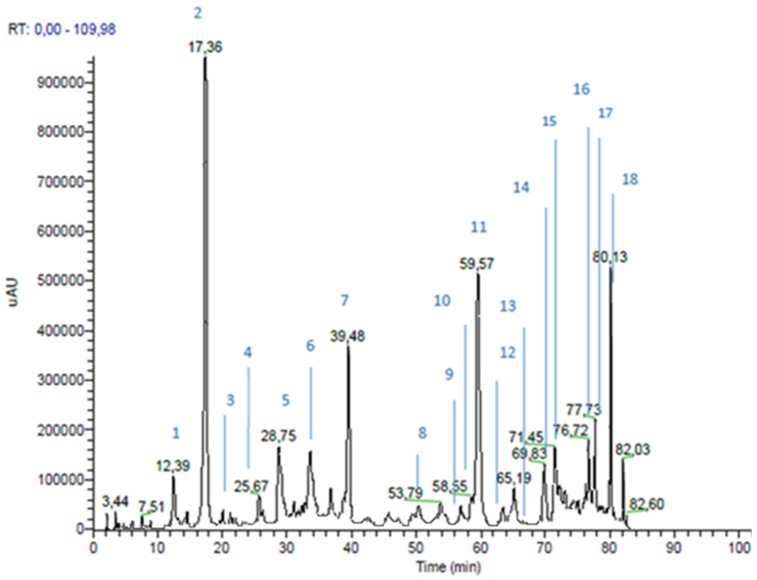
Green propolis extract chromatogram, registered at 330 nm.

**Figure 2:**
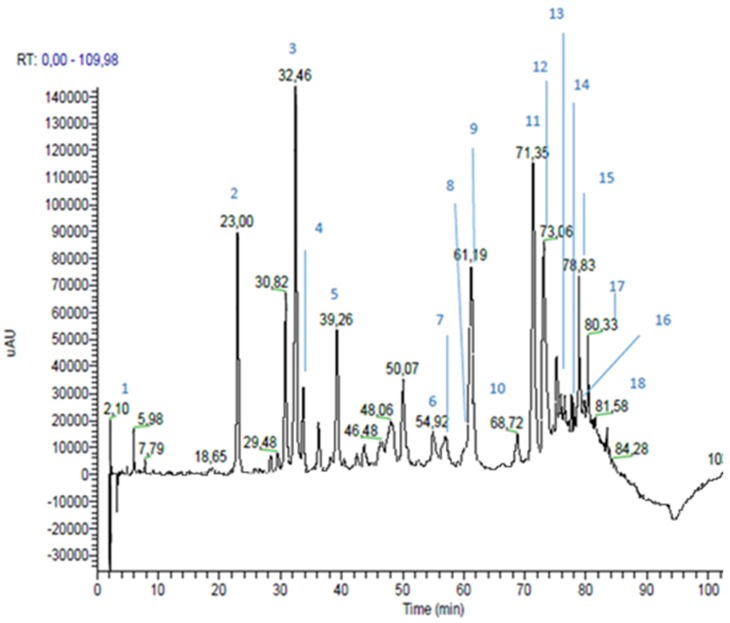
Brown propolis extract chromatogram, registered at 330 nm.

Identification was performed through the comparison of experimental data (chromatographic behavior, UV-Vis, MS and MSn spectra) with the literature, and with commercially available standard compounds, where possible. Figure 1 and Figure 2 show green and brown propolis extract chromatograms, acquired at 330 nm.

In the green propolis, six hydroxycinnamic and cinnamic acid derivatives and 11 flavonoids were identified; the percentages of the sum of their peak areas were 30.5% and 20.8%, respectively. In brown propolis, two hydroxycinnamic acids, 13 flavonoids, and one phenolic acid were identified. In contrast to green propolis, in brown propolis the major components were found to be flavonoids (sum of peak area 32.4%), followed by phenolic acids (peak area 8.5%) and hydroxycinnamic acid derivatives (sum of peak area 6.4%). Among the flavonoids, brown propolis showed a higher content of flavonols and dyhydroflavonols and a content of flavones greater than double those determined in green propolis. These results are in agreement with the results reported by similar studies. In fact, in green propolis, p-coumaric acid and artepillin C are reported to be typical components of green Brazilian propolis [29]. As far as European brown propolis is concerned, flavonoids are the major components, with flavones (i.e., chrysin and apigenin), flavanones (pinocembrin) and flavonols (galangin) being the most common brown propolis components [30].

### 3.2. Cell Viability Test

For the determination of propolis non-cytotoxic concentrations, MTT assays were performed with increasing concentrations of propolis extracts, ranging from 0.19 to 25 mg/mL, for 24 h. The highest non–cytotoxic concentration that did not cause a decrease in cell viability greater than 30%, was 3.125 mg/mL. Thus, HaCat cells were treated with 0.78, 1.56 and 3.125 mg/mL of propolis extracts for 24 h.

### 3.3. miRNA

**Figure 3:**
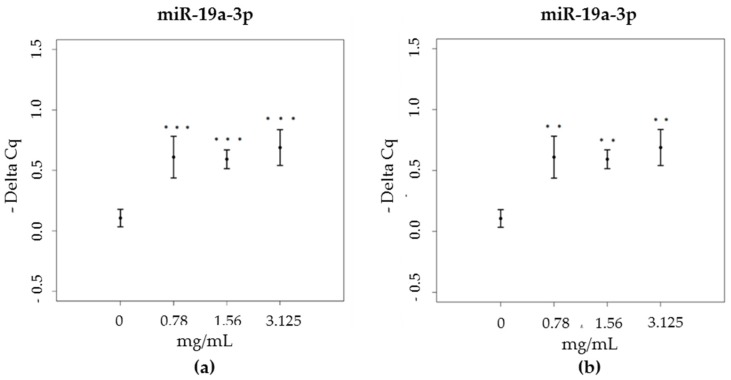
miR-19a-3p expression levels (expressed as difference of Cq – Delta-Cq) in HaCat cells, treated with increasing concentrations of (**a**) green propolis extract (0.78–3.125 mg/mL) and (**b**) brown propolis extract (0.78–3.125 mg/mL).

RNA was extracted from treated and untreated (control sample) cell cultures for subsequent RT-PCR assays. The results indicated that miR-19a-3p and miR-203a-3p, which target mRNA coding for TNF-α, were significantly upregulated by propolis. In particular, a significant increase in the expression levels of miR-19a-3p was registered following treatment with all tested concentrations of both green and brown propolis (green propolis: χ^2^ = 17.56, df = 3, *p* < 0.001; brown propolis: χ^2^ = 13.27, df = 3, *p* = 0.004), when compared to the control sample (Figure 3).

**Figure 4:**
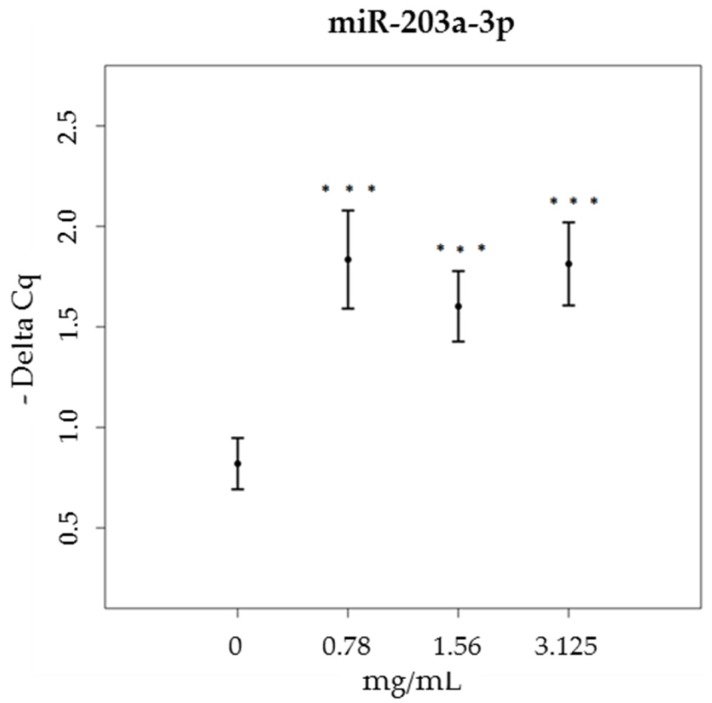
miR-203a-3p expression levels (-Delta Cq) in HaCat cells, treated with increasing concentrations of brown propolis extract (0.78–3.125 mg/mL).

On the other hand, the levels of miR-203a-3p only increased in cell cultures treated with brown propolis, at all tested concentrations (χ^2^ = 41.92, df = 3, *p* < 0.001), when compared to the control sample (Figure 4). Green propolis did not induce any significant changes in the expression level of miR-203a-3p (data not shown).

**Figure 5:**
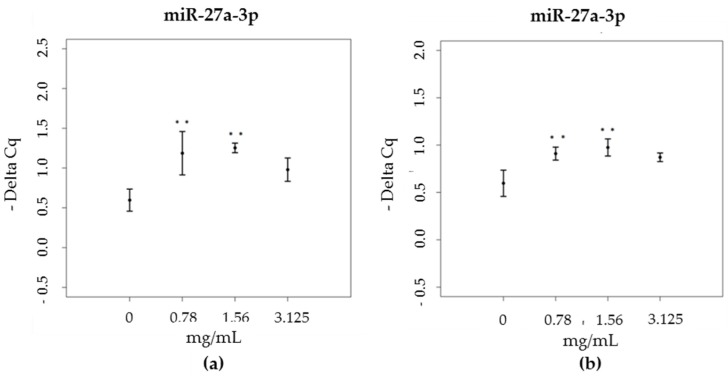
miR-27a-3p expression levels (-Delta Cq) in HaCat cells, treated with increasing concentrations of (**a**) green propolis extract (0.78–3.125 mg/mL) and (**b**) brown propolis extract (0.78–3.125 mg/mL).

As far as miR-27a-3p is concerned, it regulates NFE2L2 expression. A significant increase was registered at the two lowest concentrations for both green and brown propolis treatments (green propolis: χ^2^ = 11.28, df = 3, *p* = 0.01; brown propolis: χ^2^ = 12.90, df = 3, *p* = 0.004), compared to the control sample (Figure 5).

**Figure 6:**
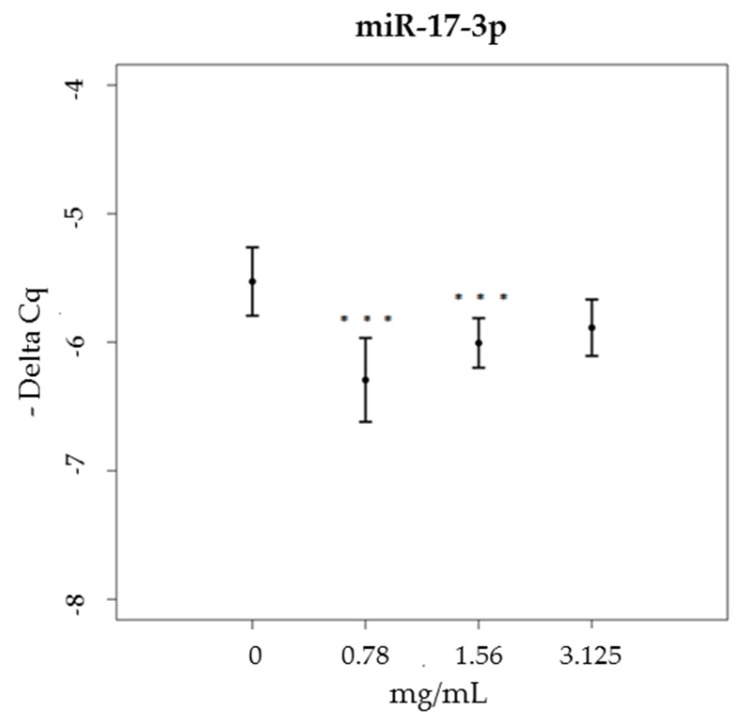
miR-17-3p expression levels (-Delta Cq) in HaCat cells, treated with increasing concentrations of brown propolis extract (0.78–3.125 mg/mL).

The expression levels of another miRNA, miR-17-3p, which targets mRNA coding for three mitochondrial antioxidant enzymes—GPX2, MnSOD and TRXR2—were significantly decreased only by brown propolis treatments at the two lowest concentrations tested (χ^2^ = 25.63, df = 3, *p* < 0.001), compared to the control sample (Figure 6, data not shown for miR-17-3p expression levels of green propolis treated cells).

### 3.4. mRNA and Proteins

**Figure 7:**
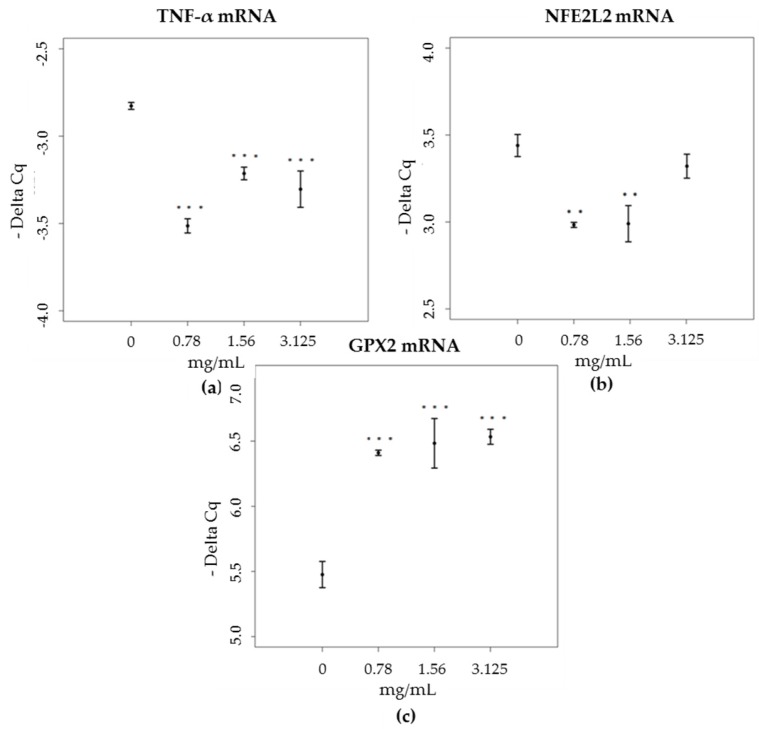
Expression levels (-Delta Cq) of mRNA coding for (**a**) TNF-α, in HaCat cells, treated with increasing concentrations of brown propolis extract (0.78–3.125 mg/mL), (**b**) NFe2L2, in HaCat cells, treated with increasing concentrations of brown propolis extract (0.78–3.125 mg/mL), (**c**) GPX2, in HaCat cells, treated with increasing concentrations of brown propolis extract (0.78–3.125 mg/mL).

The determination of the expression levels of mRNAs and proteins—which are validated targets for the studied miRNAs—was performed. For miR-19a-3p and miR-203a-3p, we investigated changes in the expression levels of mRNA coding for TNF-α. As expected, brown propolis was found to induce a decrease in the expression levels of mRNA in all cultures treated, when compared to the control sample (*F* = 16.181, *p* < 0.001; Tukey, *p* < 0.05) (Figure 7a). Conversely, green propolis did not induce any significant changes in mRNAs coding for TNF-α (data not shown). These results suggest that to decrease the expression levels of mRNAs coding for TNF-α, an increase in both the miRNAs, 19a-3p and 203a-3p, is needed.

**Figure 8:**
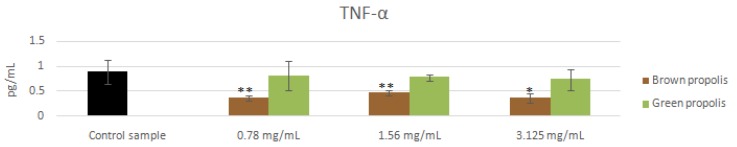
TNF-α levels in HaCat cells treated with increasing concentrations of brown propolis extract (0.78–3.125 mg/mL). * Indicates statistically significant differences (*p* < 0.05) between treated and untreated cell cultures as reported in the text. ** Indicates statistically significant differences (*p* < 0.01) between treated and untreated cell cultures as reported in the text.

TNF-α protein concentrations confirmed the expression levels of mRNAs. Significant decrease in expression levels were measured at all tested concentrations for brown propolis (*F* = 6.7292, *p* < 0.05), compared to the control sample (Figure 8). For the green propolis treatments, TNF-α concentrations did not change significantly, which also correlates with the mRNA expression levels registered (Figure 8).

For miR-27a-3p, we studied changes in the expression levels of mRNA coding for NFE2L2. As expected, we found that mRNA expression levels dropped for the two lowest concentrations in cells treated with brown propolis, in response to the overexpression of miR-27a-3p at these concentrations (*F* = 4.406, *p* < 0.05) (Figure 7b). Green propolis did not induce any significant changes in mRNAs coding for NFE2L2 (data not shown).

**Figure 9:**
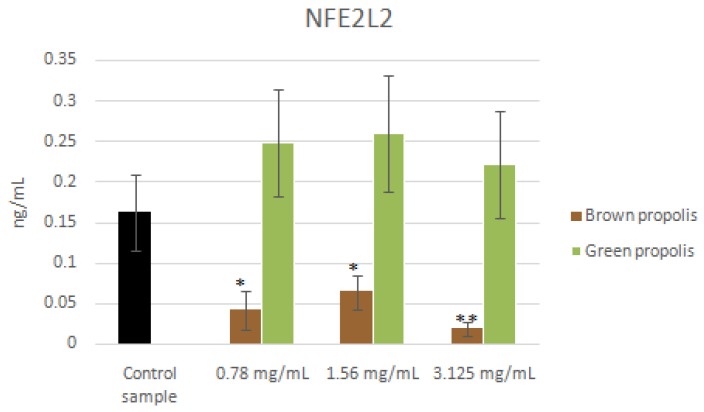
NFE2L2 levels in HaCat cells treated with increasing concentrations of brown propolis extract (0.78–3.125 mg/mL). * Indicates statistically significant differences (*p* < 0.05) between treated and untreated cell cultures as reported in the text. ** Indicates statistically significant differences (*p* < 0.01) between treated and untreated cell cultures as reported in the text.

As far as NFE2L2 is concerned, brown propolis treatment induced a decrease in the concentration of the protein in HaCat cells at all concentrations tested (*F* = 9.4892, *p* < 0.05). In agreement with the mRNA expression levels (Figure 9), the green propolis treatment did not generate any significant changes in the concentration level of the protein, compared to the control sample (Figure 9).

For the mRNA targets of miR-17-3p, involved in the regulation of mitochondrial antioxidant enzymes, namely MnSOD, GPX2 and TRXR2, the mRNAs coding for GPX2 were the only ones showing significant increases, and then only in cells treated with brown propolis, and at all concentrations tested (*F* = 20.228, *p* <0.001; Tukey, *p* ≤ 0.001), which agrees with the expression trends of the corresponding miRNA (Figure 7c).

Brown propolis treatment did not induce any significant changes in GPX2 concentrations compared to the control sample. These results suggest that GPX2 synthesis is regulated by molecular mechanisms which have not been taken into account in this study.

## 4. Discussion

Anti-inflammatory activity is one of the most studied properties of propolis. Many investigations have been performed in recent years on the effects of propolis on inflammation, in both in vitro and in vivo conditions, though the mechanism is still unclear at the molecular level.

In a model system mimicking physiological conditions, this investigation has found that brown propolis exerts anti-inflammatory activity through an epigenetic mechanism of action, being able to increase the expression levels of miR-19a-3p and miR-203a-3p, downregulate mRNA coding for TNF-α and downregulate TNF-α itself—a well-known proinflammatory cytokine involved in the initiation and propagation phases of inflammatory response—by the induction of Nuclear Factor kB (NF-kB), which is in turn involved in many biological processes, such as inflammation, immunity, differentiation, cell growth, tumorigenesis and apoptosis [31]. To the best of our knowledge, no studies have previously been performed on the anti-inflammatory activity of brown propolis on cell cultures under physiological conditions. In fact, studies performed thus far on the anti-inflammatory activity of brown propolis were carried out in model systems, in which inflammation was induced by pro-inflammatory agents [32,33,34,35]. Our results, therefore, are the first to show brown propolis exerting anti-inflammatory activity in physiological conditions and decreasing the expression of a pro-inflammatory cytokine through an epigenetic mechanism of action. This result suggests that brown propolis exerts a protective effect in healthy subjects, avoiding the development of chronic inflammation, which is a common pathological basis for many diseases, including cardiovascular disease, diabetes, and cancer.

Moreover, our investigation showed that green propolis increases the expression levels of miR-19a-3p, but does not significantly modify mRNA and TNF-α expression levels. These results are in agreement with those obtained by Kathleen et al. in 2014, who investigated TNF-α in both inflamed and non-inflamed cell cultures (mouse odontoblast-like cells, MDPC-23; macrophages, RAW264.7 and osteoclasts). They confirmed that green propolis was able to reduce TNF-α in inflamed cell cultures, as shown by other previous investigations [36,37,38,39,40], but was unable to influence TNF-α levels of cells grown in physiological conditions.

To investigate the influence of propolis on oxidative stress, we first studied the NFE2L2 transcription factor, which is encoded by a mRNA, including miR-27a-3p as a validated target. NFE2L2 is a member of the “basic leucine zipper protein” family, which regulates the transcription of genes that contain the antioxidant response element (ARE) as part of their promoter sequence; many of these genes code for proteins involved in the response to damage induced by oxidative stress and inflammation. Under physiological conditions, NFE2L2 is localized in cell cytoplasm, where the Keap1 protein mediates its transfer and degradation [41]. Oxidative stress promotes the dissociation of Keap1 from NFE2L2, which translocates into the nucleus when freed, there activating the transcription of antioxidant genes. Thus, an increase in NFE2L2 levels is associated with oxidative stress. In our experimental conditions, brown propolis was found to increase the expression levels of miR-27a-3p, confirming that brown propolis exerts an epigenetic effect. As expected, brown propolis decreased the expression levels of mRNAs coding for NFE2L2 and NFE2L2. Therefore, brown propolis acts by attenuating an oxidative stress marker in the physiological conditions applied.

This result was consistent with that obtained for the expression levels of miR-17-3p, which is involved in the regulation of mitochondrial antioxidant enzymes, namely MnSOD, GPX2 and TRXR2. Mitochondrial antioxidant defenses are responsible for the prevention of damage to cells, caused by free radicals produced by mitochondrial metabolism. The decrease of miR-17-3p after treatment with brown propolis confirms that brown propolis has the capacity to modulate miRNAs involved in protection against oxidative stress. Nevertheless, brown propolis increased the expression levels of mRNAs coding for GPX2, but did not modify the expression levels of this antioxidant enzyme itself, suggesting that the process of GPX2 synthesis is regulated by other molecular mechanisms and that no GPX2 level increase is induced in physiological conditions. In addition, brown propolis did not show any influence on mRNAs coding for the other mitochondrial enzymes, MnSOD and TRXR2. 

On the whole, these results are consistent with those obtained in different conditions by Zhang et al. which showed that brown propolis exerts radical scavengering and reducing activities, and is able to induce the nuclear translocation of NFE2L2, which, in turn, can activate the translation of antioxidant genes and phase II detoxication genes, such as HO-1 and GCLM [42].

Green propolis only increased miR-27-3p expression levels and did not induce any modification in miR-17-3p or their mRNAs and related proteins.

The different capacities to modulate the expression levels of miRNAs, mRNAs and proteins involved in the anti-inflammatory response and antioxidant activity, shown by brown and green propolis, can be ascribed to the different polyphenolic profiles of these types of propolis. The most notable difference in the chemical compositions of brown and green propolis is the higher content of flavonoids found in brown propolis, relative to hydroxycinnamic acid derivatives. This difference could be at the basis of the different behaviors. In particular, brown propolis showed higher levels of chrysin and apingenin. A large body of evidence suggests that flavones exert anti-inflammatory and antioxidant activities [43]. A recent study showed that chrysin reduced the levels of TNF-α and other pro-inflammatory cytokines and increased the activity of antioxidant enzymes in in vivo conditions (Sprague–Dawley type male rats) [44]. Similar results were achieved in a recent investigation, where, in in vitro conditions (RAW-264.7 cell line), it was shown that apigenin reduces TNF-α expression and secretion [45]. In addition, apigenin was found to improve the loss of antioxidant enzymes in vitro, exerting its activity at gene transcription, protein expression, and enzyme activity levels. Another compound which is abundant in brown propolis, in comparison with green propolis, is the flavanone, pinocembrin. In in vitro conditions (hBMEC cell line), pinocembrin regulated the NF-κB signal pathway and inhibited the release of pro-inflammatory cytokines, although it was not able to ameliorate the oxidative stress induced by cell treatment with toxic molecules, such as amyloid-β peptides [46]. On the other hand, considering the complexity of propolis, it must be highlighted that probably all these substances as a whole are responsible for the higher activity of the brown propolis. 

An in vivo investigation on experimental animals is currently ongoing to verify the effects of brown propolis, which showed more promising results than green propolis, against oxidative stress and inflammation.

## 5. Conclusions

Based on the results of this research, the antioxidant and anti-inflammatory effects attributed to green and brown propolis could be due to modulation of the levels of certain miRNAs. An interesting aspect lies in the different capacities, shown by the two types of propolis tested, to induce changes in the expression levels of miRNAs. Brown propolis, which is richer in flavonoids than in hydrocinnamic acid derivatives, was active on all miRNAs tested, while the treatment with green propolis caused changes in the expression levels of only two of the miRNAs, miR-19a-3p and miR-27a-3p. These results could suggest that brown propolis has greater epigenetic activity, probably due to the higher contents of flavanone and flavone. The same considerations can be made with regards to their ability to induce changes in the expression levels of mRNAs. In this case, brown propolis has also been shown to possess a superior modulatory capacity; it is able to modify the expression levels of mRNAs coding for TNF-α, NFE2L2, GPX2 and TNF-α and NFE2L2 protein levels.

## Figures and Tables

**Table 1:** RP-HPLC-PDA-ESI-MSn analysis elution method.

Time (min)	% Eluent A	% Eluent B
0	85	15
30	60	40
65	45	55
70	38	62
85	0	100
90	0	100
100	85	15
110	85	15

**Table 2:** Identified compounds in green propolis by RP-HPLC-PDA-ESI-MSn analyses, registered at 330 nm.

Peak *n*	RT * (min)	*m*/*z*	MS^2^ *m*/*z*		Proposed Structure	Area %
1	12.39	179	135		caffeic acid	2.4
2	17.36	163	119		p-coumaric acid	10.5
3	20.12	193	149; 134		ferulic acid	0.2
4	25.67	287	269		dihydrokaempferol	0.4
5	28.75	515	353		dicaffeoylquinic acid	2.6
6	37.21	271	253; 165; 151; 243		pinobanksin	3.4
7	39.48	301	283; 255; 215; 187		not identified	
8	53.79	247	203; 204		not identified	
9	57.02	255	213; 151		pinocembrin	0.2
10	58.55	285	257		kaempferol	2.4
11	59.57	231	187		drupanin	7.2
12	63.01	313	253; 271		pinobanksin-3-*O*-acetate	0.4
13	65.96	253	209		Chrysin	0.2
14	69.83	315	300; 271		quercetin-3-methyl-ether or quercetin-4-methyl-ether	2.8
15	71.45	299	284		luteolin-methyl-ether	3.4
16	76.72	315	300; 271		quercetin-3-methyl-ether or quercetin-4-methyl-ether	3.6
17	77.73	329	314; 299		quercetin-dimethyl-ether	4.0
18	80.13	299	200; 255		artepillin C	7.4
**Cinnamic Acid Derivatives**						**30.5**
**Flavonoids (Area 20.8%)**				**Flavonols and Dihydroflavonols**		**17.0**
				**Flavones and Flavanone**		**3.8**

* RT = retention time.

**Table 3:** Identified compounds in brown propolis by RP-HPLC-PDA-ESI-MSn analyses, registered at 330 nm.

Peak *n*	RT * (min)	*m*/*z*	MS^2^ *m*/*z*		Proposed Structure	Area %
1	2.10	225 (179 and 46)	179		caffeic acid	1.1
2	23.00	193	193; 149; 134		ferulic acid	6.3
3	32.46	137	93		p-hydroxybenzoic acid	8.5
4	33.76	285	241; 257		kaempferol	1.9
5	39.26	271	253; 243; 151; 165; 107; 225		pinobanksin	3.7
6	54.92	269	117; 149; 225		apigenin	1.8
7	56.04	315	300; 228		quercetin-3-methyl-ether	0.7
8	59.98	329	314; 299; 285		quercetin-dimethyl-ether	0.6
9	61.19	255	213; 187; 151		pinocembrin	5.1
10	68.72	313	253; 271; 299		pinobanksin-3-*O*-acetate	1.0
11	71.35	253	209		chrisin	7.8
12	73.06	269 (538)	227		galangin	7.1
13	75.20	283	239; 268		galangin-5-methyl-ether	1.1
14	76.48	327	253; 271		pinobanksin-3-*O*-propionate	0.9
15	78.83	373	279; 161; 277; 256; 305; 258		not identified	
16	79.71	341	271; 253		pinobanksin-3-*O*-butyrate	0.5
17	80.33	389	295		not identified	
18	81.58	355	253; 271; 225		pinobanksin-3-*O*-pentanoate	0.2
**Hydroxycinnamic Acids**						**6.4**
**Flavonoids (Area 32.4%)**				**Flavonoids and Dihydroflavonoids**		**22.8**
				**Flavones**		**9.6**
**Phenolic Acid**						**8.5**

* RT = retention time.

## References

[1] Marcucci M.C. (1995). Propolis: Chemical composition, biological properties and therapeutic activity. Apidologie.

[2] Attia Y.A., Abd Al-Hamid A.E., Ibrahim M.S., Al-Harthi M.A., Bovera F., Elnaggar A.S. (2014). Productive performance, biochemical and hematological traits of broiler chickens supplemented with propolis, bee pollen, and mannan oligosaccharides continuously or intermittently. Livest. Sci..

[3] Lotfy M. (2006). Biological activity of bee propolis in health and disease. Asian Pac. J. Cancer Prev..

[4] Cvek J., Medid-Saric M., Vitali D., Vedrina-Dragojevik I., Smit Z., Tomic S. (2008). The content of essential and toxic elements in Croatian propolis samples and their tinctures. J. Apicult. Res..

[5] Kuropatnicki A.K., Szliszka E., Krol W. (2013). Historical aspects of propolis research in modern times. Evid. Based Complement. Altern. Med..

[6] Liu C.F., Lin C.C., Lin M.H., Lin Y.S., Lin S.C. (2002). Cytoprotection by propolis ethanol extract of acute absolute ethanol-induced gastric mucosal lesions. Am. J. Chin. Med..

[7] Barros M.P., Lemos M., Maistro E.L., Leite M.F., Sousa J.P., Bastos J.K., Andrade S.F. (2008). Evaluation of antiulcer activity of the main phenolic acids found in Brazilian green propolis. J. Ethnopharmacol..

[8] Casaroto A.R., Lara V.S. (2010). Phytomedicines for Candida-associated denture stomatitis. Fitoterapia.

[9] Marinotti S., Ranzato E. (2015). Propolis: A new frontier for wound healing?. Burns Trauma.

[10] Kurek-Gorecka A., Rzepecka-Stojko A., Gorecki M., Stpiko J., Sosada M., Swierczek-Zieba G. (2013). Structure and antioxidant activity of polyphenols derived from propolis. Molecules.

[11] Abo-Salem O.M., El-Edel R.H., Harisa G.E.I., Halawany N.E., Ghonaim M.M. (2009). Experimental diabetic nephropathy can be prevented by propolis: Effect on metabolic disturbance and renal oxidative parameters. Pakistan J. Pharm. Sci..

[12] Hu F., Zhu W., Chen M., Shou Q., Li Y. (2011). Biological activities of Chinese propolis and Brazilian propolis on streptozotocin-induced type 1 diabetes mellitus in rat. Evid. Based Complement. Altern. Med..

[13] Wang T., Chen L., Wu W., Long Y., Wang R. (2008). Potential cytoprotection: Antioxidant defence by caffeic acid phenethyl ester against free radical-induced damage of lipids, DNA, and proteins. Can. J. Physiol. Pharmacol..

[14] Chee H.Y. (2002). In vitro evaluation of the antifungal activity of propolis extract on Cryptococcus neoformans and Candida albicans. Microbiology.

[15] Banskota A.H., Tezuka Y., Adnyana I.K., Midorikawa K., Matsushige K., Message D., Huertas A.A., Kadota S. (2000). Cytotoxic, hepatoprotective and free radical scavenging effects of propolis from Brazil, Peru, the Netherlands and China. J. Ethnopharmacol..

[16] Lahouel M., Boulkour S., Segueni N., Fillastre J.P. (2004). The flavonoids effect against vinblastine, cyclophosphamide and paracetamol toxicity by inhibition of lipid-peroxidation and increasing liver glutathione concentration. Pathol. Biol..

[17] Natarajan K., Singh S., Burke T.R., Grunberger D., Aggarwal B.B. (1996). Caffeic acid phenethyl ester is a potent and specific inhibitor of activation of nuclear transcription factor NF-kappa B. Proc. Natl. Acad. Sci. USA.

[18] Rajoo M., Parolia A., Pau A., Amalraj F.D. (2004). The role of propolis in inflammation and orofacial pain: A review. Ann. Res. Rev. Biol..

[19] Bartel D.P. (2004). MicroRNAs: Genomics, biogenesis, mechanism, and function. Cell.

[20] Kumazaki M. (2014). Propolis cinnamic acid derivatives induce apoptosis through both extrinsic and intrinsic apoptosis signaling pathways and modulate of miRNA expression. Phytomedicine.

[21] Cuevas A., Saavedra N., Cavalcante M.F., Salazar L.A., Abdalla D.S. (2014). Identification of microRNAs involved in the modulation of pro-angiogenic factors in atherosclerosis by a polyphenol-rich extract from propolis. Arch. Biochem. Biophys..

[22] Cuevas A., Saavedra N., Rudnicki M., Abdalla D.S.P., Salazar L.A. (2015). ERK1/2 and HIF1α are involved in antiangiogenic effect of polyphenols-enriched fraction from Chilean propolis. Evid. Based Complement. Altern. Med..

[23] Park Y.K., Alencar S.M., Aguiar C.L. (2002). Botanical origin and chemical composition of Brazilian propolis. J. Agric. Food Chem..

[24] Huang S., Zhang C.P., Wang K., Li G.Q., Hu F.L. (2014). Recent advances in the chemical composition of propolis. Molecules.

[25] Volpi N., Fachini A. Procedimento Per L’ottenimento di Estratti Integrali di Propoli Ricchi in Polifenoli e Dotati di Attività Antibatterica e Sua Applicazione Nella Prevenzione e Trattamento di Processi Infettivi di Origine Batterica.

[26] Zhang C., Huang S., Wei W., Ping S., Shen X., Li Y., Hu F. (2014). Development of high-performance liquid chromatographic for quality and authenticity control of Chinese propolis. J. Food Sci..

[27] Curti V., Di Lorenzo A., Rossi D., Martino E., Capelli E., Collina S., Daglia M. (2017). Enantioselective modulatory effects of naringenin enantiomers on the expression levels of miR-17-3p involved in endogenous antioxidant defenses. Nutrients.

[28] Curti V., Capelli E., Boschi F., Nabavi S.F., Bongiorno A.I., Habtemariam S., Nabavi S.M., Daglia M. (2014). Modulation of human miR-17-3p expression by methyl 3-O-methyl gallate as explanation of its in vivo protective activities. Mol. Nutr. Food Res..

[29] Machado B.A., Silva R.P., Barreto Gde A., Costa S.S., Silva D.F., Brandão H.N., Rocha J.L., Dellagostin O.A., Henriques J.A., Umsza-Guez M.A. (2016). Chemical composition and biological activity of extracts obtained by supercritical extraction and ethanolic extraction of brown, green and red propolis derived from different geographic regions in Brazil. PLoS ONE.

[30] Volpi N., Bergonzini G. (2006). Analysis of flavonoids from propolis by on-line HPLC-electrospray mass spectrometry. J. Pharm. Biomed. Anal..

[31] Chang R., Yee K.L., Sumbria R.K. (2017). Tumor necrosis factor α inhibition for Alzheimer’s disease. J. Cent. Nerv. Syst. Dis..

[32] Wang K., Zhang J., Ping S., Ma Q., Chen X., Xuan H., Shi J., Zhang C., Hu F. (2014). Anti-inflammatory effects of ethanol extracts of Chinese propolis and buds from poplar (*Poplus × canadensis*). J. Ethnopharmacol..

[33] Bufalo C.M., Bordon-Graciani A.P., Conti B.J., Golim M.A., Sforcin J.M. (2014). The immunomodulatory effect of propolis on receptors expression, cytokine production and fungicidal activity of human monocytes. J. Pharm. Pharmacol..

[34] Khayyal M.T., El-Hazek R.M., El-Ghazaly M.A. (2015). Propolis aqueous extract preserves functional integrity of murine intestinal mucosa after exposure to ionizing radiation. Environ. Toxicol. Pharmacol..

[35] Ertürküner S.P., Saraç E.Y., Göçmez S.S., Ekmekçi H., Öztürk Z.B., Seçkin I., Sever Ö., Keskinbora K. (2016). Anti-inflammatory and ultrastructural effects of Turkish propolis in a rat model of endotoxin-induced uveitis. Folia Histochem. Cytobiol..

[36] Wu Z., Zhu A., Takayama F., Okada R., Liu Y., Harada Y., Wu S., Nakanishi H. (2013). Brazilian green propolis suppresses the hypoxia-induced neuroinfammatory responses by inhibiting NF-kB activation in microglia. Oxid. Med. Cell. Longev..

[37] Takeshita T., Watanabe W., Toyama S., Hayashi Y., Honda S., Sakamoto S., Matsuoka S., Yoshida H., Takeda S., Hidaka M. (2013). Effect of Brazilian propolis on exacerbation of respiratory syncytial virus infection in mice exposed to tetrabromobisphenol A, a brominated flame retardant. Evid. Based Complement. Altern. Med..

[38] Neiva K.G., Catalfamo D.L., Holliday S., Wallet S.M., Pileggi R. (2014). Propolis decreases lipopolysaccharide-induced inflammatory mediators in pulp cells and osteoclast. Dent. Traumatol..

[39] Zhao L., Pu L., Wei J., Li J., Wu J., Xin Z., Gao W., Guo C. (2016). Brazilian green propolis improves antioxidant function in patients with type 2 diabetes mellitus. Int. J. Environ. Res. Public Health.

[40] Tiveron A.P., Rosalen P.L., Franchin M., Lacerda R.C.C., Bueno-Silva B., Benso B., Denny C., Ikegaki M., de Alencar S.M. (2016). Chemical characterization and antioxidant, antimicrobial, and anti-inflammatory activities of south Brazilian organic propolis. PLoS ONE.

[41] Chen B., Lu Y., Chen Y., Cheng J. (2015). The role of Nrf2 in oxidative stress-induced endothelial injuries. J. Endocrinol..

[42] Zhang L., Shen X., Wang K., Cao X., Zhang C., Zheng H., Hu F. (2016). Antioxidant activities and molecular mechanisms of the ethanol extracts of Baccharis popolis and Eucalyptus propolis in RAW64.7 cells. Pharm. Biol..

[43] Nabavi S.F., Braidy N., Habtemariam S., Orhan I.E., Daglia M., Manayi A., Gortzi O., Nabavi S.M. (2015). Neuroprotective effects of chrysin: From chemistry to medicine. Neurochem. Int..

[44] Eldutar E., Kandemir F.M., Kucukler S., Caglayan C. (2017). Restorative effects of Chrysin pretreatment on oxidant-antioxidant status, inflammatory cytokine production, and apoptotic and autophagic markers in acute paracetamol-induced hepatotoxicity in rats: An experimental and biochemical study. J. Biochem. Mol. Toxicol..

[45] Palacz-Wrobel M., Borkowska P., Paul-Samojedny M., Kowalczyk M., Fila-Danilow A., Suchanek-Raif R., Kowalski J. (2017). Effect of apigenin, kaempferol and resveratrol on the gene expression and protein secretion of tumor necrosis factor alpha (TNF-α) and interleukin-10 (IL-10) in RAW-264.7 macrophages. Biomed. Pharmacother..

[46] Rui L., Li J.-Z., Song J.-K., Sun J.-L., Li Y.-J., Zhou S.-B., Zhang T.-T., Du G.-H. (2014). Pinocembrin protects human brain microvascular endothelial cells against fibrillar Amyloid-β1−40 injury by suppressing the MAPK/NF-κB inflammatory pathways. Biomed. Res. Int..

